# Breath Analysis of Propofol and Associated Metabolic Signatures: A Pilot Study Using Secondary Electrospray Ionization–High-resolution Mass Spectrometry

**DOI:** 10.1097/ALN.0000000000005531

**Published:** 2025-04-21

**Authors:** Jiafa Zeng, Nikola Stankovic, Kapil Dev Singh, Regula Steiner, Urs Frey, Thomas Erb, Pablo Sinues

**Affiliations:** 1 Department of Biomedical Engineering, University of Basel, Basel, Switzerland; Department of Pulmonology, University Children’s Hospital Basel, Basel, Switzerland; First Affiliated Hospital of Jinan University, Guangzhou, China.; 2 Department of Anesthesia, University Children’s Hospital Basel, Basel, Switzerland.; 3 Department of Biomedical Engineering, University of Basel, Basel, Switzerland; Department of Pulmonology, University Children’s Hospital Basel, Basel, Switzerland.; 4 Institute for Clinical Chemistry, University Hospital Zurich, Zurich, Switzerland.; 5 Department of Biomedical Engineering, University of Basel, Basel, Switzerland; Department of Pulmonology, University Children’s Hospital Basel, Basel, Switzerland.; 6 Department of Anesthesia, University Children’s Hospital Basel, Basel, Switzerland.; 7 Department of Biomedical Engineering, University of Basel, Basel, Switzerland; Department of Pulmonology, University Children’s Hospital Basel, Basel, Switzerland.

## Abstract

**Background:**

Propofol is a widely used anesthetic for total IV anesthesia. Although it is generally safe, rare but serious complications can occur in vulnerable groups, such as critically ill patients and children. Clinicians often rely on surrogate measures (*e.g.*, predicted effect-site concentrations or Bispectral Index), yet more direct indicators of anesthetic exposure and metabolic stress would be valuable. The authors hypothesized that pharmacometabolomics *via* breath analysis could yield real-time insights into propofol concentrations as well as accompanying metabolic responses to surgery.

**Methods:**

In this pilot study, 10 pediatric patients (median age, 5.9 yr; interquartile range, 4.3 to 6.6) undergoing propofol anesthesia contributed 47 breath samples (10 preinduction, 37 postinduction) and 37 blood samples. All samples were analyzed by high-resolution mass spectrometry. Linear mixed-effects models examined associations between exhaled compounds and serum propofol concentrations while accounting for repeated measures in individual patients. Volcano plots were used to identify differential changes in metabolites after propofol induction.

**Results:**

Propofol, its metabolites, and endogenous metabolites were readily detected in exhaled breath, demonstrating strong correlations with serum propofol concentrations (partial *R*² ≥ 0.65; adjusted *P* < 0.001). Differential analysis showed significant upregulation of endogenous fatty aldehydes (log_2_ [postinduction/preinduction] ≥ 1; adjusted *P* ≤ 0.05), suggestive of lipid peroxidation and oxidative stress. Exogenous compounds, including benzene and phenols, were also observed, reflecting propofol metabolism *in vivo*.

**Conclusions:**

This pilot study highlights a robust breath–serum relationship for propofol and reveals surgery-associated shifts in metabolic pathways, including evidence of oxidative stress. These findings underscore the feasibility of exhaled-breath pharmacometabolomics for individualized anesthetic care. Further validation in larger cohorts is warranted to confirm clinical utility and to determine whether real-time breath analysis could ultimately serve as a useful adjunct for guiding anesthetic management and monitoring perioperative metabolic responses.

## Abstract 2

This pilot study in children having propofol-based anesthesia found that on‐site secondary electrospray ionization–high-resolution mass spectrometry breath pharmacometabolomics can capture robust correlations between exhaled signals and serum propofol concentrations while revealing significant metabolic shifts likely linked to oxidative stress.

## Editor’s Perspective

**What We Already Know about This Topic**

- During total IV anesthesia with propofol, direct blood concentration measurement is not routinely achievable, and thus, clinicians rely on surrogate measures such as predicted effect-site concentrations or Bispectral Index
- Breath analysis by secondary electrospray ionization–high-resolution mass spectrometry is emerging as a noninvasive metabolomics technique that may be applicable to clinical contexts

**What This Article Tells Us That Is New**

- This pilot study in children having propofol-based anesthesia found that on-site secondary electrospray ionization–high-resolution mass spectrometry breath pharmacometabolomics can capture robust correlations between exhaled signals and serum propofol concentrations while revealing significant metabolic shifts likely linked to oxidative stress

Propofol is one of the most widely used anesthetics in operating rooms and intensive care units due to its favorable pharmacokinetics, rapid reversibility, and established safety profile. Its metabolism primarily occurs in the liver, undergoing conjugation and hydroxylation pathways, followed by glucuronidation or sulfation.^1,2^ Additionally, the lungs contribute approximately 30% of propofol uptake and first-pass elimination after a bolus dose.^3^ Genetic variations, age, and pathologic conditions can influence propofol metabolism and clearance, leading to interindividual variability.^4–6^ In pediatric patients, propofol clearance differs significantly. For example, clearance rates in neonates have been reported to be only 10 to 38% of adult values, with further changes occurring during organ development.^7^ Although advanced target-controlled infusion (TCI) models (*e.g.*, Eleveld) incorporate age-related maturation, dosing in neonates and young children may still benefit from additional refinements to account for patient-specific factors, such as genetic variability or disease states.

In clinical practice, TCI is increasingly utilized for propofol, with covariates like age, weight, height, and sex used to improve dosing precision.^8–10^ Nonetheless, pediatric pharmacokinetics can exhibit a higher degree of variability.^11–13^ Approaches offering real-time feedback on anesthetic exposure, such as monitoring blood concentrations of propofol or its metabolites, may provide valuable supplemental guidance, especially during total IV anesthesia (TIVA). However, conventional blood sampling is invasive, time-consuming, and not well suited for immediate feedback in routine clinical settings.

Propofol, along with some of its metabolites, can also be detected in exhaled breath due to their volatility.^14–18^ Breath analysis by secondary electrospray ionization–high-resolution mass spectrometry (SESI-HRMS) is emerging as a noninvasive metabolomics technique in clinical contexts. SESI-HRMS offers high sensitivity and specificity, capturing a broad metabolic range and showing promise for therapeutic drug monitoring and patient stratification in response to medication in clinical settings, including pediatrics.^19–22^

This pilot study adopts a comprehensive metabolomic strategy in pediatric patients undergoing TIVA, capturing not only propofol and its metabolites but also a wide range of endogenous metabolites whose levels shift in response to anesthesia and surgery. By correlating serum propofol concentrations with exhaled breath profiles *via* SESI-HRMS, we aimed to map the broader metabolic cascade triggered by the procedure. We hypothesize that tracking endogenous metabolic changes can provide additional clinical insights beyond traditional therapeutic drug monitoring by offering real-time information on both anesthetic exposure and the physiologic impact of surgical stress.

## Materials and Methods

### Study Design and Patient Recruitment

The study was part of the study of Exhaled Breath Analysis by Secondary Electrospray Ionization–Mass Spectrometry in Children and Adolescents (EBECA), ClinicalTrials.gov ID NCT04461821. The study was approved by the Ethics Committee of North-Western and Central Switzerland (Basel, Switzerland; 2020-00778; more information can be found at https://clinicaltrials.gov/study/NCT04461821?term=pablo%20sinues&rank=1) and conducted at the University Children’s Hospital Basel (Basel, Switzerland) in compliance with Good Clinical Practice and the Declaration of Helsinki. This pilot study recruited 10 pediatric patients undergoing TIVA for dental surgery. All participants were free of comorbidities and additional medications and were classified as American Society of Anesthesiologists (Schaumburg, Illinois) Physical Status I or II. Written informed consent was obtained at least 24 h before the procedure from the parents or legal guardians.

**Fig. 1.**
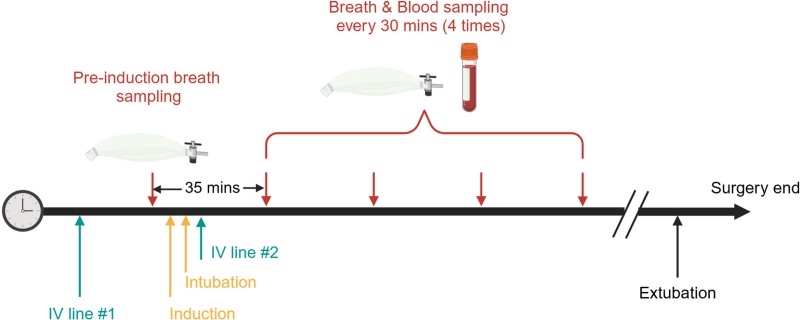
Schematic workflow during surgery. Breath samples were collected using Nalophan bags (Scentroid, Canada), while venous blood samples (0.5 ml each) were drawn in parallel. Preinduction breath samples were taken 5 min before induction, followed by four additional paired breath and blood samples collected at 30-min intervals after induction. The timeline includes key procedural steps, including intravenous (IV) line placements, induction, intubation, and sampling, up to the end of surgery.

Parallel sampling of exhaled breath and venous blood was performed in the operating room. Preinduction breath sampling was conducted approximately 5 min before induction, during spontaneous breathing, using Nalophan bags (Scentroid, Canada) for subsequent offline breath analysis, as previously described.^23,24^ All 10 patients were premedicated orally with midazolam at a dose of approximately 0.3 mg/kg. In patients who underwent IV induction, lidocaine at the dose of 1 mg/kg was administered into a peripheral vein with proximal tourniquet applied 1 min before the start of the propofol TCI bolus. At the discretion of the anesthesiologist in charge, five patients were relaxed with rocuronium. In 4 of the 10 patients, an IV catheter (for drug administration) was placed after local analgesia (Eutectic Mixture of Local Anesthetics, Aspen Pharma Schweiz GmbH, Switzerland) on the back of the hand. Propofol was administered *via* TCI using the PAedfusor model^25^ (Alaris PK pump, CareFusion, United Kingdom), alongside a bolus of remifentanil (0.33 μg · kg^–1^ · min^–1^). In the remaining six patients, inhalational induction with sevoflurane was performed due to needle phobia, after which an IV line was placed, followed by propofol and remifentanil administration. The administered medication is reported in detail in Supplemental Digital Content table S1 (https://links.lww.com/ALN/D979). All patients were orotracheally intubated, and a second IV catheter (for blood sampling) was placed on the dorsum of the foot. The first paired breath (maximum, 2 l) and blood (0.5 ml) samples were collected 30 min after induction. Three additional sample sets were collected at 30-min intervals. Sampling workflow details are depicted in figure 1, and the breath sampling procedure is outlined in Supplemental Digital Content figure S1 (https://links.lww.com/ALN/D974).

Once the exhaled breath was collected in the sampling bag, it was analyzed in 3 min using SESI-HRMS (SuperSESI and SuperSESI-X, Fossil Ion Tech, Spain; Q-Exactive Plus and Orbitrap Exploris 240, Thermo Fisher Scientific, Germany). The sampling bag was directly deflated into the mass spectrometer for real-time analysis. Propofol and its reported metabolites^26^ were identified based on accurate mass-to-charge ratios and further confirmed using propofol standards (Pharmaceutical Secondary Standard, Merck, Germany) by matching their tandem mass spectrometry spectra (Supplemental Digital Content fig. S2, https://links.lww.com/ALN/D975). Blood samples were centrifuged within 30 min after collection, and the serum was stored at –80°C for subsequent analysis. Total propofol concentrations in serum were measured at the Institute for Clinical Chemistry, University Hospital Zürich (Zürich, Switzerland), using high-performance liquid chromatography coupled with tandem high-resolution mass spectrometry (Q-Exactive, Thermo Fisher Scientific). Measurements of unbound propofol concentrations could not be performed due to laboratory capacity limitations.

### Statistical Analysis

Detailed preprocessing procedures for the mass-spectrometric data were performed as described previously.^27^ Briefly, the acquired *.RAW files were processed using in-house C# console (Microsoft, USA) applications based on Thermo Fisher Scientific’s RawFileReader (version 5.0.0.38) and MATLAB (version R2022a; MathWorks Inc., USA). During the preparation of this work, the authors used ChatGPT o1 Pro to streamline the MATLAB code used for the analysis. After using this tool/service, the authors reviewed and edited the content as needed. Representative mass spectra were obtained for each sample by averaging scans collected during the analysis window. Mass spectra were calibrated to achieve a mass accuracy of ±1 ppm, allowing for precise molecular formula assignments. To reduce noise, artifact satellite peaks were removed *via* apodization. Features were binned at ±1 ppm using MATLAB’s *ksdensity* function, resulting in 6,008 features (4,001 in positive mode, 2,007 in negative mode).

Postprocessing retained only features present (*i.e.*, signal intensity > 0) in at least 60% of postinduction samples, reducing the matrix to 1,322 features (958 positive, 364 negative). Zeros (16% of all data points) were imputed using the regression on order statistics method^28^ in R (https://www.r-project.org/; Version 4.1.2; accessed November 1, 2021). Subsequent log_10_ transformation resulted in a data distribution close to a normal distribution. To account for batch effects arising from the two different mass spectrometers (SuperSESI plus Q-Exactive Plus *vs*. SuperSESI-X plus Orbitrap Exploris 240) and sevoflurane exposure as a known covariate, we applied the ComBat method.^29,30^

Because the same patients were measured multiple times (repeated measures), we used linear mixed-effects modeling^31,32^ to appropriately account for the correlated structure within each patient. After fixing this structure, we estimated fixed-effect slopes for each feature, testing their association with serum propofol concentration using false discovery rate–adjusted *P* values. To further balance the effect size against variability, we computed a partial *R*² statistic for each feature’s slope (reflecting proportion of variance explained). Significant features were ranked by partial *R*² to prioritize robust effect sizes. The top features (ranked by partial *R*²) were visualized with individual trajectories, population-level regression lines, and 95% confidence bands to quantify biomarker–propofol relationships.

To identify significantly altered metabolic features, paired *t* tests were performed comparing preinduction and postinduction breath signal intensities. *P* values were adjusted for multiple comparisons using Storey’s q-value method.^33^ Features meeting the thresholds log_2_(postinduction/preinduction) ≥ 1 and *q* ≤ 0.05 were classified as significantly altered and associated with the intervention.

Compound mapping of accurate masses in the database of RefMet: A Reference List of Metabolite Names^34^ was conducted using customized R script based on the MetaboAnalystR package.^35^ Then matched compound names were further cross-referenced with the Human Metabolome Database, PubChem, and Kyoto Encyclopedia of Genes and Genomes database.

## Results

### Detection of Propofol and Associated Metabolites in Exhaled Breath

A total of 47 breath samples and 37 blood samples were collected from 10 pediatric patients. Ten of the 47 breath samples corresponded to preinduction measurements, where blood concentrations of propofol were assumed to be zero. Table 1 lists the characteristics of the recruited pediatric patients. All breath measurements were completed without any disruption of the routine surgical procedures.

**Table 1.** Characteristics of Examined Patients

Patients, n	Age, yr	Sex	Weight, kg
10	5.9 (4.3–6.6)	50% Female	18.8 (17.7–26.0)

Data presented as median (interquartile range).

**Fig. 2.**
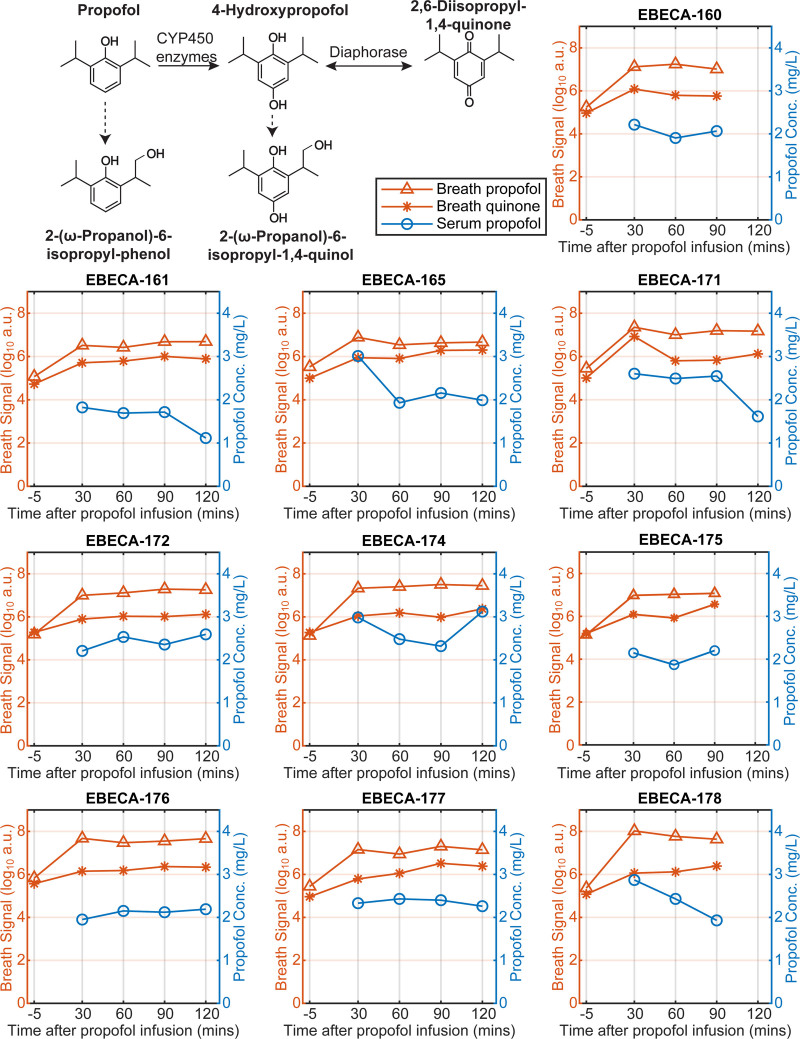
Schematic of propofol metabolic transformations. (*Top left*) Propofol undergoes metabolism primarily *via* cytochrome P450 (CYP450) enzymes, leading to the formation of 4-hydroxypropofol, which can be further oxidized to 2,6-diisopropyl-1,4-quinone *via* diaphorase. *Dashed arrows* indicate minor metabolic routes. Time traces (*bottom panels*) depict signal intensities corresponding to protonated propofol (*orange triangles*) and protonated 2,6-diisopropyl-1,4-quinone (*orange stars*) detected in the breath samples for each patient. Serum propofol concentrations (Conc.; *blue circles*) are shown for reference. Breath signals in the preinduction samples correspond to background chemical noise. Please note the log_10_ scale of the breath signal intensities. a.u., arbitrary unit; EBECA, Exhaled Breath Analysis by Secondary Electrospray Ionization–Mass Spectrometry in Children and Adolescents; EBECA-xxx, patient ID.

Figure 2 (top left) illustrates a simplified version of the propofol metabolic pathway. Propofol (2,6-diisopropylphenol, C_12_H_18_O) is first metabolized by cytochrome P450 enzymes, forming 4-hydroxypropofol (C_12_H_18_O_2_), which can be further oxidized to 2,6-diisopropyl-1,4-quinone (C_12_H_16_O_2_) chemically or *via* diaphorase, establishing a tautomeric equilibrium. Minor metabolic pathways can yield 2-(ω-propanol)-6-isopropyl-phenol (C_12_H_18_O_2_) and 2-(ω-propanol)-6-isopropyl-1,4-quinol (C_12_H_18_O_3_). Targeted analysis of mass-to-charge ratio peaks associated with these metabolites showed that both propofol and its metabolite 2,6-diisopropyl-1,4-quinone were clearly detectable in exhaled breath after propofol infusion. Figure 2 displays the signal intensities of protonated propofol and 2,6-diisopropyl-1,4-quinone in exhaled breath as a function of time, alongside the corresponding serum propofol concentrations. Preinduction breath signal intensities above zero were consistent with the presence of background chemical noise (*i.e.*, presence of isomeric species).

Interestingly, 2,6-diisopropyl-1,4-quinone has also been reported as an impurity in propofol formulations. To distinguish between its metabolic origin and potential formulation impurities, we analyzed the headspace of the propofol formulation using SESI-HRMS. Signals corresponding to propofol and 2,6-diisopropyl-1,4-quinone were detectable, but the ratio of these signals was significantly higher in patients’ breath than in the formulation (17% *vs.* 2%; *P* = 0.005; Supplemental Digital Content fig. S3, https://links.lww.com/ALN/D976). This suggests that, while part of the observed signal could originate from impurities, the majority likely results from hepatic metabolism.

Two additional signals potentially associated with propofol metabolites (4-hydroxypropofol and 2-(ω-propanol)-6-isopropyl-1,4-quinol) were detected in negative ion mode. However, due to ion suppression from sevoflurane in six patients (Supplemental Digital Content fig. S4, https://links.lww.com/ALN/D977), these data were not further analyzed. Ion suppression was not observed in positive ion mode, so subsequent analyses focused exclusively on positive ions.

### Associations between Serum Propofol and Breath Features

**Fig. 3.**
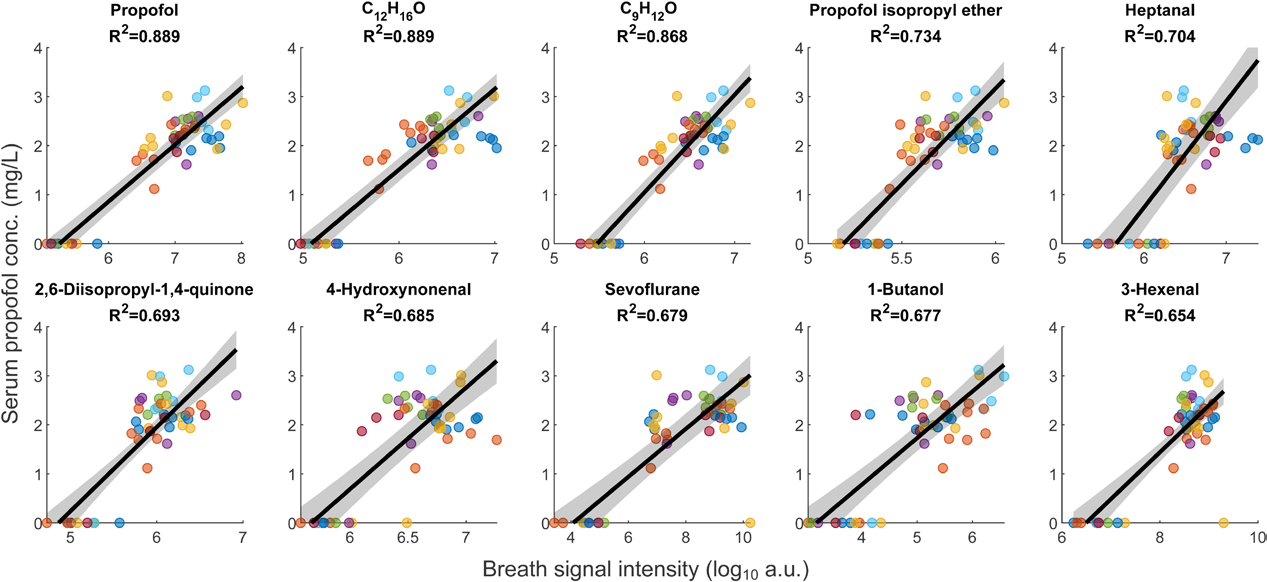
Top 10 breath-derived features most strongly associated with serum propofol concentration (conc.; partial *R*^2^ > 0.65) under a linear mixed-effects framework. Each plots log_10_-transformed breath-feature intensities (*horizontal axis*) *versus* measured serum propofol concentrations (*vertical axis*) for multiple measurements from individual patients (*colored circles*). The *black solid line* represents the population-level level linear mixed-effects fit, while the *shaded region* denotes the 95% CI for that fitted line. The resulting partial *R*^2^ value for each displayed feature is shown *above each plot*. a.u., arbitrary unit.

After our targeted analysis, we used linear mixed-effects modeling to examine how serum propofol concentrations relate to the various breath features. We ranked significant features by their partial *R*² values and excluded isotopes (*i.e.*, redundant peaks) and features lacking an assignable molecular formula. This yielded 51 features with partial *R*² ≥ 0.30 and false discovery rate–adjusted *P* values ≤ 0.05 (Supplemental Digital Content table S2, https://links.lww.com/ALN/D980). Figure 3 highlights the top 10 breath features, each exhibiting a strong association (*R*² ≥ 0.65) with serum propofol. As expected, exhaled propofol showed the highest partial *R*² (0.889), closely followed by a propofol-related compound (C_12_H_16_O) with the same value. Two other propofol-related metabolites, C_9_H_12_O and propofol isopropyl ether (C_15_H_24_O), had partial *R*² values of 0.868 and 0.734, respectively. Meanwhile, 2,6-diisopropyl-1,4-quinone remained substantial at 0.693. Four endogenous molecules—heptanal, 4-hydroxynonenal, 1-butanol, and 3-hexenal—also displayed robust partial *R*² values (0.654 to 0.704) and were identified as fatty alcohols and aldehydes, meriting further discussion in the next section. Notably, sevoflurane exhibited a partial *R*² of 0.679, indicating a similarly strong relationship with serum propofol concentrations.

### Surgery-induced Metabolic Cascade

**Fig. 4.**
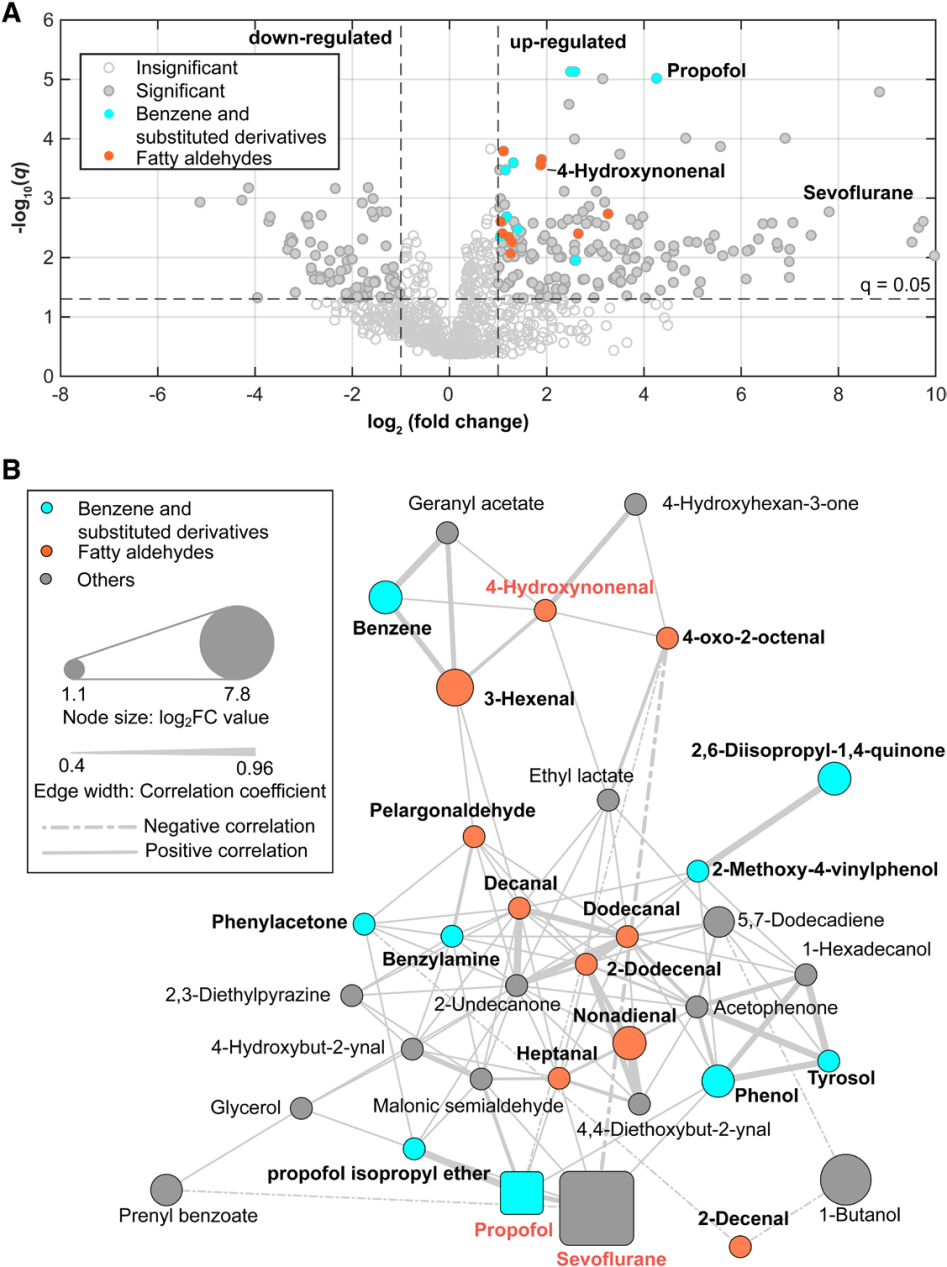
Metabolic cascade induced during surgery under TIVA. (*A*) Volcano plot: A total of 173 significantly upregulated breath features (log_2_ fold change ≥ 1 and *q* ≤ 0.05; *gray dots, right*) and 78 significantly downregulated features (log_2_ fold change ≤ –1 and *q* ≤ 0.05; *gray dots, left*) were identified. Additionally, specific chemical groups were highlighted: 9 benzene and substituted derivatives (*cyan dots*) and 10 aldehydes (*orange dots*). (*B*) Correlation network: This network comprises 34 identified upregulated compounds, organized into three chemical groups: benzene and substituted derivatives (9 compounds, *cyan nodes*), aldehydes (10 compounds, *orange nodes*), and others (15 compounds, *gray nodes*). *Node size* represents the log_2_ fold change values (ranging from 1.1 to 7.8), while *edge width* reflects the strength of the correlation (absolute *r* values between 0.4 and 0.96). *Solid edges* indicate positive correlations, and *dashed edges* indicate negative correlations. Propofol and sevoflurane are distinctively represented as *square nodes*.

After the identification of a strong association between serum propofol and a subset of exhaled molecules (exogenous and endogenous), metabolic changes in exhaled breath induced by propofol and overall the surgical intervention were investigated by analyzing the changes in signal intensities across the entire suit of molecules detected in exhaled breath. Among the 958 features detected in positive ion mode, 367 features showed significant differences (*P* ≤ 0.05) between pre- and postinduction samples based on paired *t* tests of breath signal intensities. After applying false discovery rate correction, 349 features retained significance (q ≤ 0.05). Supplemental Digital Content fig. S5 (https://links.lww.com/ALN/D978) illustrates the p- and q-value distributions of all positive ion mode features. The log_2_ fold change (log_2_FC) was calculated for each feature to quantify the magnitude of change. Figure 4A, a volcano plot, shows that 173 features were significantly upregulated (log_2_FC ≥ 1; *q* ≤ 0.05), while 78 features were significantly downregulated (log_2_FC ≤ −1; *q* ≤ 0.05) after propofol induction. Among the upregulated features, 35 were identified at the compound level by querying databases, including propofol, its metabolite 2,6-diisopropyl-1,4-quinone, sevoflurane, and various endogenous metabolites. Details of these altered compounds are summarized in Supplemental Digital Content table S3 (https://links.lww.com/ALN/D981). To gain further insights into the relationships among the 35 identified compounds, a correlation network was constructed based on pairwise correlation coefficients (fig. 4B). This analysis revealed two distinct chemical groups: (1) fatty aldehydes, the largest group, comprising 10 compounds; and (2) benzene and substituted derivatives, consisting of 9 compounds, including propofol and its metabolite. The remaining compounds were categorized as “others.” One compound, 2-cyanopyridine, was excluded from the network due to its low connectivity (absolute *r* < 0.4) with other compounds.

## Discussion

Pharmacometabolomics leverages metabolic profiles to predict individual drug responses, thereby advancing precision medicine. Unlike static pharmacogenomics, it offers dynamic insights influenced by diet, the microbiome, disease states, and environmental factors. Proof-of-principle studies have shown its utility in personalizing treatments—for instance, predicting acetaminophen toxicity or stratifying statin responders—thereby guiding dose adjustments, minimizing adverse events, and refining therapeutic strategies.^36–39^ This approach is particularly valuable for managing the interindividual variability observed in vulnerable populations, such as pediatric or critically ill patients, where standard pharmacokinetic models can face greater challenges. It also captures rapid metabolic fluctuations, as often seen with anesthetics whose clinical effects emerge within minutes. With ongoing advances in high-resolution mass spectrometry and data analytics, pharmacometabolomics holds promise for enhancing drug monitoring and therapeutic decisions. Recently, we demonstrated its feasibility in pediatric patients receiving antiseizure^21^ and bronchodilator^40^ medications by investigating the exhaled metabolome—an approach especially suitable for children due to its noninvasive nature. In this study, we sought to extend this breath-based pharmacometabolomics strategy to monitor propofol, its metabolites, and associated endogenous metabolic shifts during a routine clinical intervention.

Propofol remains one of the most frequently administered anesthetics in operating rooms and intensive care units, owing to its favorable pharmacokinetic profile, rapid reversibility, and well-established safety record. Nonetheless, tailoring its dosage to specific patient groups—particularly children and critically ill individuals—can be complex. Direct measurement of propofol at the actual site of action (*e.g.*, the brain) is not currently feasible in clinical practice; instead, clinicians rely on model-based “effect-site” predictions and electroencephalography-derived parameters as surrogate indicators of depth of anesthesia. These methods generally perform well, yet unusual complications like propofol infusion syndrome^41–43^ call for additional technology to flag such rare but potentially lethal adverse reactions, especially in children.^42^ Advanced TCI models, such as the Eleveld model, already incorporate age, weight, height, and sex to improve dosing precision. Still, genetic factors, developmental status, and pathophysiological conditions introduce interpatient variability in metabolism and clearance. Neonates, for example, may display propofol clearance rates only 10 to 38% of adult levels, with ongoing maturation affecting pharmacokinetics over time. Such variability can complicate optimal dosing decisions, highlighting the potential benefits of additional monitoring modalities in pediatric anesthesia.

Our first objective was to couple the SESI-HRMS breath metabolomics technique in a real-world operating room (fig. 1; Supplemental Digital Content fig. S1, https://links.lww.com/ALN/D974) in mechanically ventilated patients, aiming to evaluate the accuracy and utility of this noninvasive method in this environment to explore this monitoring during propofol-based anesthesia. This pilot study revealed that the collection of metabolic information could be seamlessly integrated into standard surgical workflows, causing no discomfort to either clinicians or patients. Breath samples were analyzed on-site using SESI-HRMS without additional processing, providing a mass spectral readout typically within 15 min of collection. Future integration of the mass spectrometer directly into the ventilator system could reduce this turnaround to near real-time, on the order of seconds. Indeed, a proof-of-concept for continuous breath monitoring *via* SESI-HRMS at 10-s intervals during sleep has already been demonstrated.^44^

We could also confirm that the metabolic information contained in the collection devices includes propofol and propofol metabolites along with a rich metabolic signature of endogenous compounds. An initial targeted inspection of the mass spectra revealed that propofol was clearly detected in postinduction samples and showed signal intensities that were two orders of magnitude higher than the preinduction samples. These results are consistent with previous studies showing that propofol is detectable in exhaled breath and furthermore correlates with systemic concentrations.^14,17,22,45–47^ However, one key novelty here is that we report on the simultaneous detection of propofol and one of its metabolites, 2,6-diisopropyl-1,4-quinone (fig. 2), which has been suggested to render around one third the hypnotic activity of propofol while all other metabolites are inactive.^18^ Regardless of whether propofol metabolites are pharmacologically inactive, their measurement is essential for understanding hepatic metabolism and optimizing dosing strategies. Propofol undergoes extensive liver metabolism *via* glucuronidation and hydroxylation, primarily mediated by uridine diphosphate–glucuronosyltransferase and cytochrome P450 enzymes, and altered metabolite profiles can indicate hepatic dysfunction, enzyme saturation, or interindividual variability due to genetic or pharmacologic factors.^26^ We therefore argue that monitoring metabolites can serve as an early biomarker for hepatic stress. In addition, the lungs play a pivotal role in the initial distribution of propofol, with a notable portion of the drug being temporarily sequestered during its first pass through the pulmonary circulation. This process influences the timing and magnitude of propofol’s central effects, highlighting the importance of considering pulmonary factors in its clinical use^3,48^ and the role that exhaled propofol could have toward this end.

Along with propofol itself (partial *R*^2^ = 0.889) and its known metabolite, 2,6-diisopropyl-1,4-quinone (partial *R*^2^ = 0.693), several additional compounds exhibited strong associations with serum propofol concentrations (partial *R*^2^ ≥ 0.65; adjusted *P* < 0.001; fig. 3). These compounds can be broadly classified into two categories: drug-related molecules and endogenous metabolites. Within the drug-related category, we identified propofol isopropyl ether, a compound documented as Propofol Related Compound C in United States Pharmacopeia (North Bethesda, Maryland) reference standards. Two additional, unidentified compounds (molecular formulae C_9_H_12_O and C_12_H_16_O) were also highly correlated with serum propofol (partial *R*^2^ = 0.868 and 0.889, respectively). Both of these formulae are plausibly related to propofol (C_12_H_18_O) through structural or metabolic modifications. Although we cannot yet determine whether these are previously unreported propofol metabolites or arise from another source, their close molecular similarity and strong association with propofol strongly suggest that they are drug-related entities.

Another exogenous compound that appeared to be associated with propofol was sevoflurane. This observation is not entirely unexpected, as propofol and sevoflurane are frequently coadministered in clinical practice, and previous studies on exhaled propofol have noted potential interference from sevoflurane.^49,50^ In our own SESI-HRMS platform, sevoflurane caused ion suppression in negative ion mode but not in positive ion mode. More puzzlingly, we observed a strong association (partial *R*² = 0.679) even in patients who did not receive sevoflurane. One plausible explanation is that trace levels of this highly volatile compound remained in the sampling setup despite thorough cleaning procedures, leading to residual carryover signals in subsequent measurements. Given the high volatility of sevoflurane and the sensitivity of the SESI-HRMS approach, these residual signals may have produced a spurious correlation with propofol levels. This highlights the need for careful consideration of potential contamination sources and rigorous quality control measures in breath-based analyses.

Finally, within the endogenous metabolites category, we found fatty alcohol (1-butanol) and fatty aldehydes (heptanal, 4-hydroxynonenal, and 3-hexenal), which are associated with oxidated stress.^51^ Further differential and correlation analysis combined with queries to metabolite databases confirmed an overall substantial metabolic alteration triggered during the surgical intervention (fig. 4; Supplemental Digital Content table S3, https://links.lww.com/ALN/D981). While this massive metabolic alteration remains to be fully characterized, given the extent of molecules involved, it is plausible that they are not only drug metabolites but also a vast majority of endogenous metabolites.^21^ Indeed, this is also consistent with previous metabolomics studies providing critical insights into the biochemical changes triggered by surgical stress, including alterations in energy metabolism, amino acid catabolism, and oxidative stress.^52–54^ These metabolic shifts reflect the body’s response to increased energy demands, muscle protein breakdown, and reactive oxygen species production during surgery. Thus, we queried our data against metabolite data sets and could tentatively annotate 35 molecules (Supplemental Digital Content table S3, https://links.lww.com/ALN/D981). Apart from propofol, we also found sevoflurane, which illustrates the capability to monitor multiple anesthetics simultaneously. The main group of endogenous metabolites identified corresponds to 10 aldehydes, including 4-hydroxynonenal, which is a well-characterized marker of oxidative stress. The generalized positive correlation across these compounds and their central positioning in the network support the notion that these metabolites are tightly regulated. Indeed, elevated aldehydes are indicative of lipid damage and oxidative imbalance, which are hallmarks of the surgical metabolic stress response. Similarly, we have recently shown that this same technique can monitor the recovery of children with diabetic ketoacidosis.^55^ Severe metabolic acidosis is one of the hallmarks of propofol infusion syndrome,^41–43^ further suggesting that the rich metabolic fingerprint accessible by this technique could potentially serve to monitor propofol concentrations but also flag potential side effects at an individual level.

Limitations of this study include the following: SESI-HRMS instrumentation requires a substantial capital investment, although a cost analysis suggests a per-sample cost (approximately $20) comparable to other clinical tests.^56^ Breath measurements may be influenced by ventilation and perfusion changes, causing a lag between exhaled and effect-site concentrations; nevertheless, multiple studies show strong correlations with serum^14–17,45–47^ and brain^57,58^ propofol levels. This feasibility study needs further validation to confirm our findings and to determine whether these exhaled molecules serve as reliable predictive biomarkers. Additional targeted mass-spectrometric analyses using chemical standards are also necessary to fully confirm metabolite identities.

In conclusion, on-site SESI-HRMS breath pharmacometabolomics can capture robust correlations between exhaled signals and serum propofol concentrations while revealing significant metabolic shifts likely linked to oxidative stress. Integrating these data with established pharmacokinetic models and electroencephalography-based measures could advance individualized anesthetic management, although larger studies are required to confirm clinical utility.

### Acknowledgments

The authors thank Dr. Fabienne Decrue, M.D., Ph.D. (Emergency Department, University Children’s Hospital Basel, Basel, Switzerland), for coordinating and assisting in the collection of samples and measurements during the initial stages of the experiment. The authors thank Isabel González Novoa (Department of Pulmonology, University Children’s Hospital Basel) and Maria Beck, M.D. (Department of Anesthesia, University Children’s Hospital Basel) for conducting the patients’ recruitment, interviews, and document filing. Mélina Richard, M.S. (Department of Biomedical Engineering, University of Basel, Basel, Switzerland), is gratefully acknowledged for coordinating the study. Schematic workflow was created with BioRender.com (BioRender, Canada). During the preparation of this work, the authors used ChatGPT 4o to improve the readability of the manuscript. After using this tool/service, the authors reviewed and edited the content as needed and take full responsibility for the content of the publication.

### Research Support

Dr. Sinues received funding from Fondation Botnar (Basel, Switzerland) and the Swiss National Science Foundation (Bern, Switzerland; PCEGP3_181300).

### Competing Interests

Dr. Sinues is a cofounder of Deep Breath Intelligence AG (Zurich, Switzerland), which develops breath-based diagnostic tools. Dr. Zeng was a part-time employee (maximum 20%, from February 2023 to February 2024) of Deep Breath Intelligence AG. Dr. Singh is a part-time employee of Deep Breath Intelligence AG. The other authors declare no competing interests.

## Supplemental Digital Content

Figure S1. Breath sampling setup, https://links.lww.com/ALN/D974

Figure S2. Tandem mass spectrometry profiles of propofol detected in breath, https://links.lww.com/ALN/D975

Figure S3. Ratio of quinone *versus* propofol signal in breath, https://links.lww.com/ALN/D976

Figure S4. Sevoflurane and three features in negative ion mode, https://links.lww.com/ALN/D977

Figure S5. Distributions of *p* and *q* value of paired *t* tests, https://links.lww.com/ALN/D978

Table S1. Patients and medications information, https://links.lww.com/ALN/D979

Table S2. Breath features correlated with serum propofol, https://links.lww.com/ALN/D980

Table S3. Information of identified compounds, https://links.lww.com/ALN/D981

## Supplementary Material
