# Supplementary material for: Breath Analysis of Propofol and Associated Metabolic Signatures: A Pilot Study Using Secondary Electrospray Ionization–High-resolution Mass Spectrometry
Source: Anesthesiology. 2025 Apr 21;143(2):345–56. doi: 10.1097/ALN.0000000000005531 (PMC12227210; doi:10.1097/ALN.0000000000005531)
Supplement: Supplementary file 1 [file aln-143-345-s001.pdf]

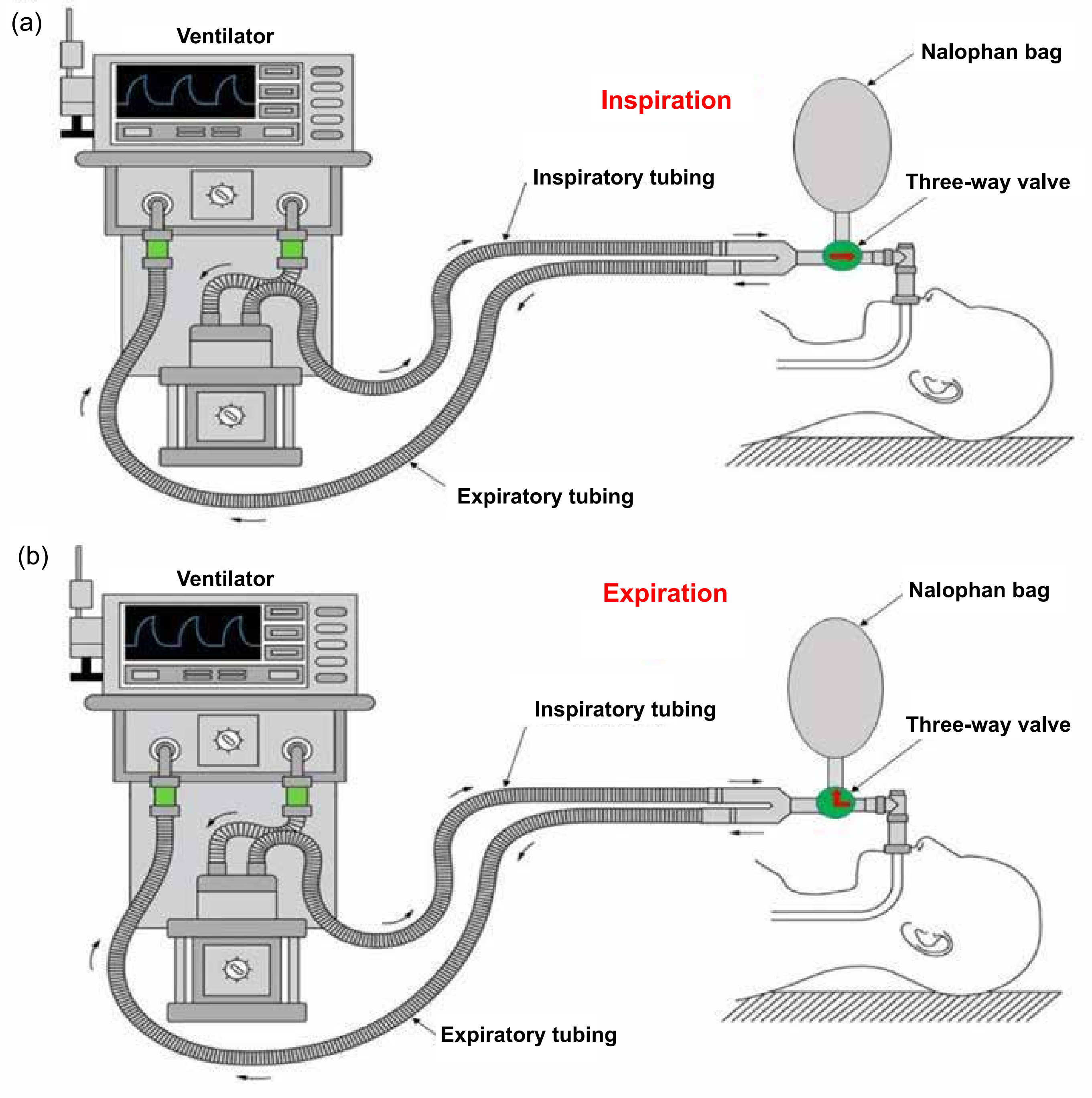
(a)
Ventilator
Inspiration
Nalophan bag
Inspiratory tubing
Three-way valve
Expiratory tubing
(b)
Ventilator
Expiration
Nalophan bag
Inspiratory tubing
Three-way valve
Expiratory tubing

Figure S1. Breath sampling method. Nalophan bags and three-way Hans-Rudolf valves were used for exhaled breath sampling. During manual ventilation the three-way valve was switched according (synchronizing) to the phase of respiratory cycle. (a) During inspiration, the valve was set manually to close the sampling bag so that flow is like in normal mechanical ventilation. (b) During expiration the valve was set manually to close the expiratory tubing and to allow flow of exhaled air to the sampling bag. The valves were cleaned and disinfected before each measurement.
