# Supplementary material for: Breath Analysis of Propofol and Associated Metabolic Signatures: A Pilot Study Using Secondary Electrospray Ionization–High-resolution Mass Spectrometry
Source: Anesthesiology. 2025 Apr 21;143(2):345–56. doi: 10.1097/ALN.0000000000005531 (PMC12227210; doi:10.1097/ALN.0000000000005531)
Supplement: Supplementary file 2 [file aln-143-345-s002.pdf]

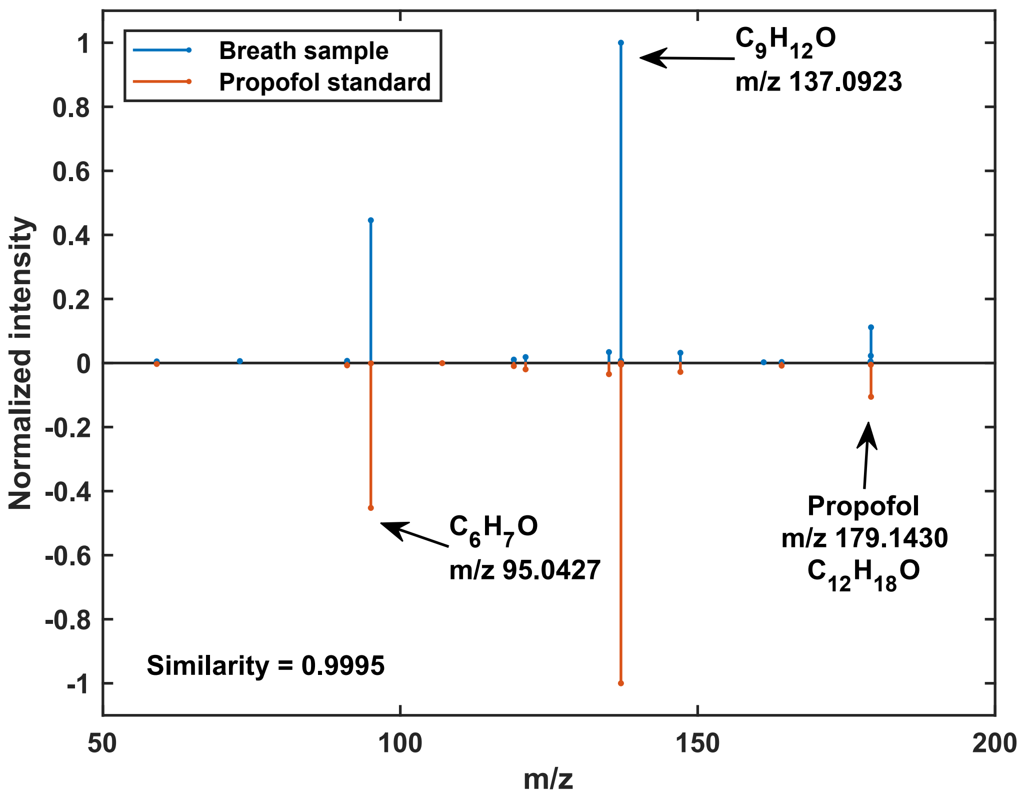

Breath sample
Propofol standard
C9H12O
m/z 137.0923
C6H7O
m/z 95.0427
Propofol
m/z 179.1430
C12H18O
Similarity = 0.9995
Normalized intensity
m/z
1
0.8
0.6
0.4
0.2
0
-0.2
-0.4
-0.6
-0.8
-1
50
100
150
200

Figure S2. MS-MS profiles of propofol detected in breath sample (on top, in blue) and standard (on bottom, in orange).
