# Supplementary material for: Breath Analysis of Propofol and Associated Metabolic Signatures: A Pilot Study Using Secondary Electrospray Ionization–High-resolution Mass Spectrometry
Source: Anesthesiology. 2025 Apr 21;143(2):345–56. doi: 10.1097/ALN.0000000000005531 (PMC12227210; doi:10.1097/ALN.0000000000005531)
Supplement: Supplementary file 3 [file aln-143-345-s003.pdf]

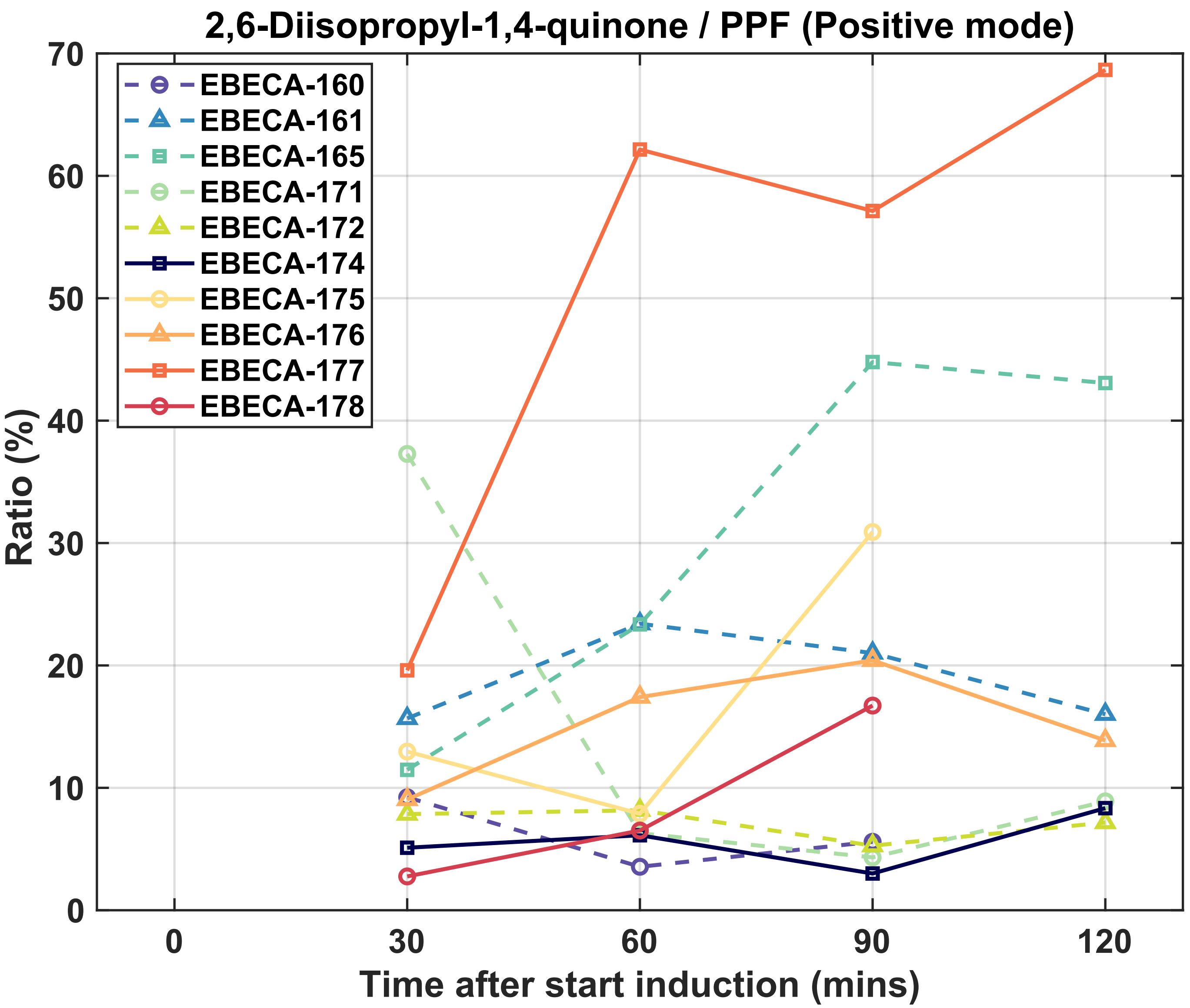

2,6-Diisopropyl-1,4-quinone / PPF (Positive mode)
EBECA-160
EBECA-161
EBECA-165
EBECA-171
EBECA-172
EBECA-174
EBECA-175
EBECA-176
EBECA-177
EBECA-178
Ratio (%)
0
10
20
30
40
50
60
70
0
30
60
90
120
Time after start induction (mins)

Figure S3. Ratio of 2,6-Diisopropyl-1,4-quinone vs. propofol breath signal for each patient.
