# Supplementary material for: Breath Analysis of Propofol and Associated Metabolic Signatures: A Pilot Study Using Secondary Electrospray Ionization–High-resolution Mass Spectrometry
Source: Anesthesiology. 2025 Apr 21;143(2):345–56. doi: 10.1097/ALN.0000000000005531 (PMC12227210; doi:10.1097/ALN.0000000000005531)
Supplement: Supplementary file 4 [file aln-143-345-s004.pdf]

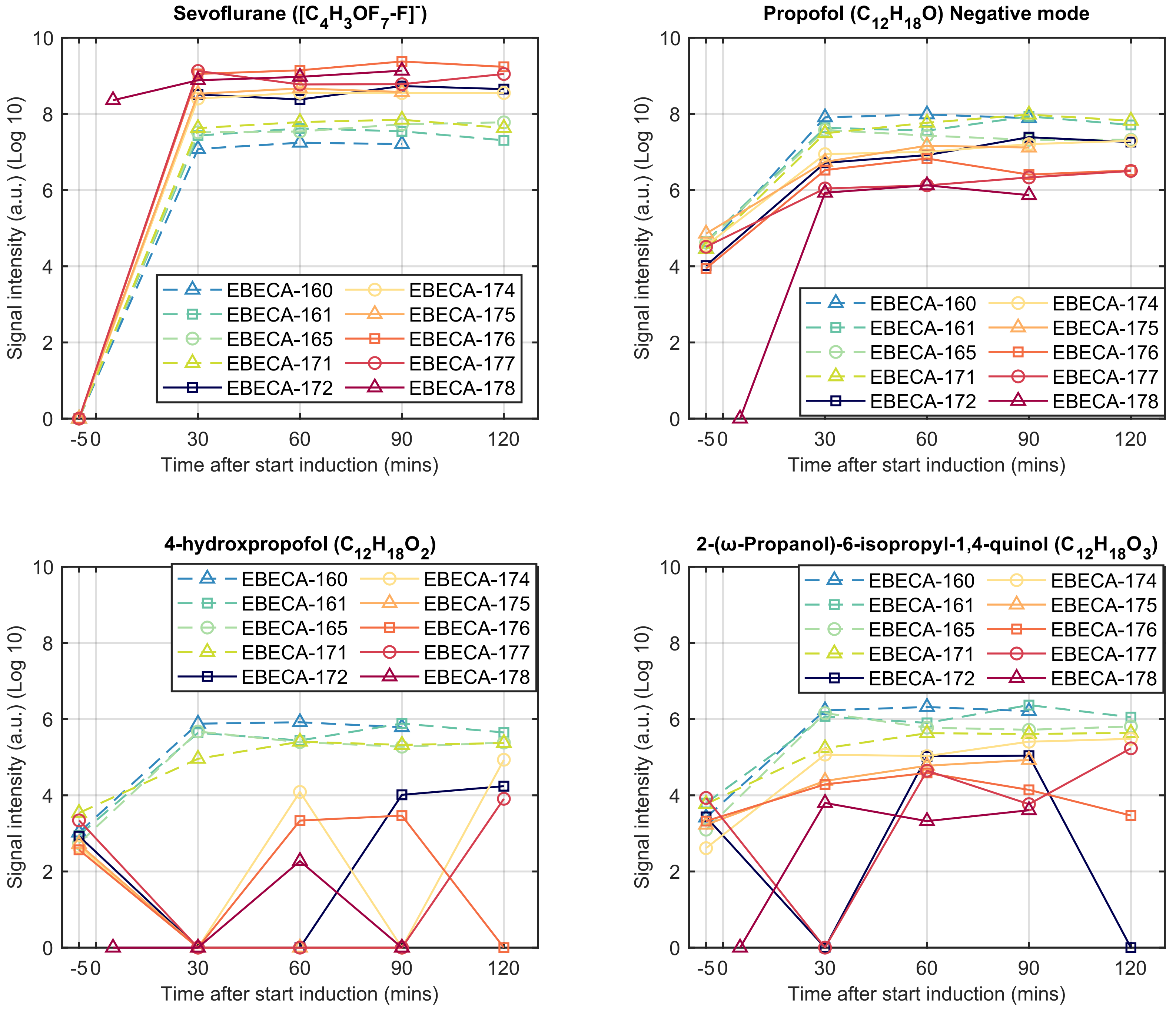

Sevoflurane ($[C_4H_3OF_7\text{-}F]^-$)
Propofol ($C_{12}H_{18}O$) Negative mode
4-hydroxpropofol ($C_{12}H_{18}O_2$)
2-(ω-Propanol)-6-isopropyl-1,4-quinol ($C_{12}H_{18}O_3$)
Signal intensity (a.u.) (Log 10)
Time after start induction (mins)
-50
30
60
90
120
0
2
4
6
8
10
EBECA-160
EBECA-161
EBECA-165
EBECA-171
EBECA-172
EBECA-174
EBECA-175
EBECA-176
EBECA-177
EBECA-178

Figure S4. Sevoflurane ([C4H3OF7-F+O2]-), propofol, 4-Hydroxpropofol and 2-(ω-Propanol)-6-isopropyl-1,4-quinol in negative ion mode. Dash line means samples without sevoflurane, solid line means samples with sevoflurane. To be noted, in the patient 178 initial breath sampling was performed after inhalational induction with sevoflurane.
