# Supplementary material for: Breath Analysis of Propofol and Associated Metabolic Signatures: A Pilot Study Using Secondary Electrospray Ionization–High-resolution Mass Spectrometry
Source: Anesthesiology. 2025 Apr 21;143(2):345–56. doi: 10.1097/ALN.0000000000005531 (PMC12227210; doi:10.1097/ALN.0000000000005531)
Supplement: Supplementary file 5 [file aln-143-345-s005.pdf]

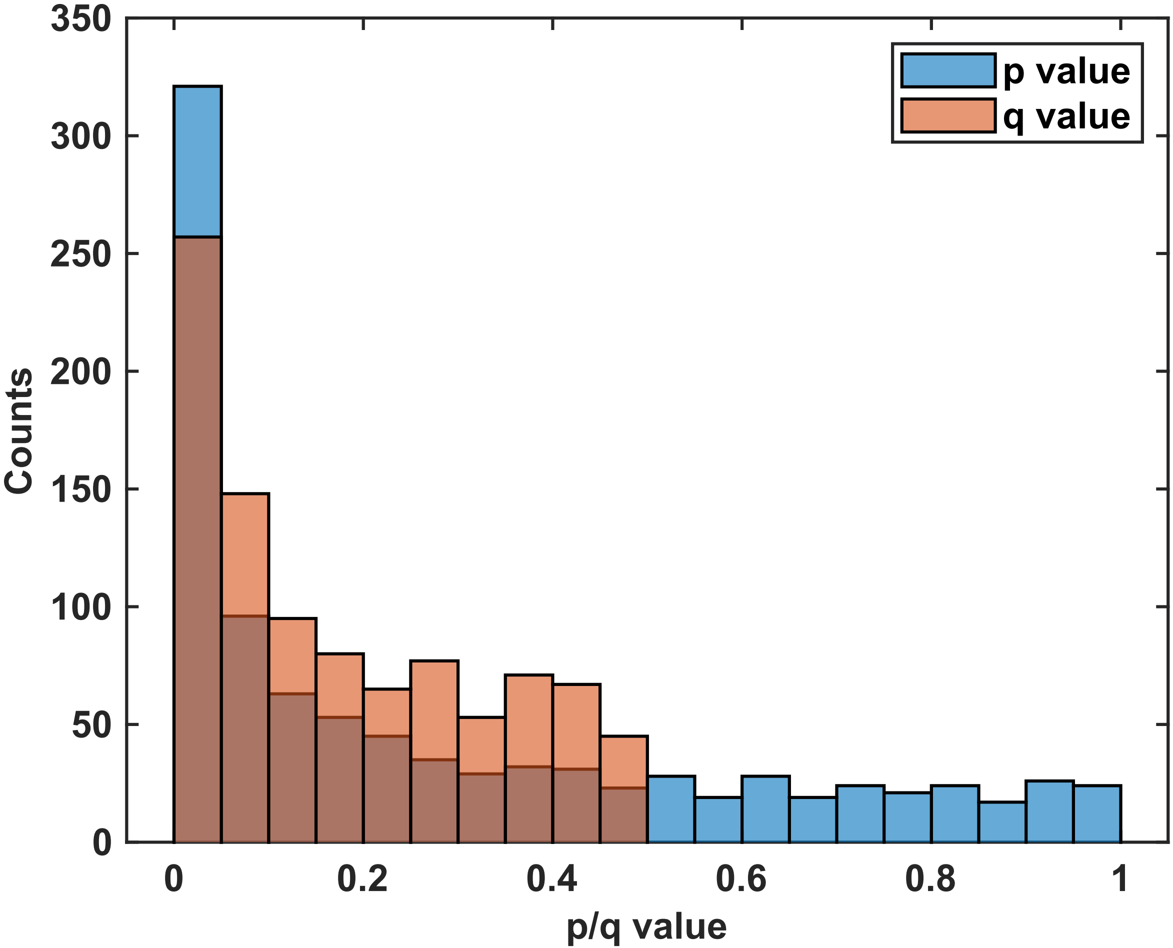

p value
q value
Counts
p/q value
0
50
100
150
200
250
300
350
0
0.2
0.4
0.6
0.8
1

Figure S5. The distributions of p- and q-value of paired t-tests (pre/post) of all breath feature in positive ion mode.
