# Supplementary material for: Breath Analysis of Propofol and Associated Metabolic Signatures: A Pilot Study Using Secondary Electrospray Ionization–High-resolution Mass Spectrometry
Source: Anesthesiology. 2025 Apr 21;143(2):345–56. doi: 10.1097/ALN.0000000000005531 (PMC12227210; doi:10.1097/ALN.0000000000005531)
Supplement: Supplementary file 6 [file aln-143-345-s006.pdf]

Table S1. Patients and medications information.

Patient ID	Gender	Age	Weight (kg)	Propofol target plasma concentration (mg/L)	Sevoflurane (%)	Remifentanil (μg/kg/min)	Midazolam (mg/kg)	Rocuronium (mg)	Lidocaine (mg/kg)
EBECA_160	Male	8.6	26.0	2	/	0.33	0.3	/	1
EBECA_161	Male	6.2	18.0	2	/	0.33	0.3	10	1
ECECA_165	Female	2.1	10.0	2	/	0.33	0.3	10	1
EBECA_171	Female	6.6	17.7	2	/	0.33	0.3	10	1
EBECA_172	Female	6.2	34.0	2	8% - 5% - 0	0.33	0.3	/	/
EBECA_174	Female	9.6	36.1	2	8% - 5% - 0	0.25	0.3	30	/
EBECA_175	Male	5.6	21.0	2	8% - 5% - 0	0.33	0.3	/	/
EBECA_176	Female	4.3	15.4	2	8% - 5% - 0	0.33	0.3	/	/
EBECA_177	Male	4.7	19.0	2	8% - 5% - 0	0.33	0.3	10	/
EBECA_178	Female	4.1	18.5	2	8% - 5% - 0	0.33	0.3	/	/
