# Supplementary material for: Breath Analysis of Propofol and Associated Metabolic Signatures: A Pilot Study Using Secondary Electrospray Ionization–High-resolution Mass Spectrometry
Source: Anesthesiology. 2025 Apr 21;143(2):345–56. doi: 10.1097/ALN.0000000000005531 (PMC12227210; doi:10.1097/ALN.0000000000005531)
Supplement: Supplementary file 7 [file aln-143-345-s007.pdf]

Table S2. Breath features correlated with serum propofol (partial R2≥0.3)

m/z	**Formula**	**Name**	**Partial R^2**	**Slope**	**p value**	**adj-p value**
179.1430	$C_{12}H_{18}O$	Propofol	0.889	1.160	3.890E-23	2.792E-20
177.1274	$C_{12}H_{16}O$	/	0.889	1.645	4.224E-23	2.792E-20
137.0961	$C_9H_{12}O$	/	0.868	1.992	2.060E-21	9.077E-19
221.1900	$C_{15}H_{24}O$	Propofol isopropyl ether	0.734	3.900	1.608E-14	1.417E-12
115.1117	$C_7H_{14}O$	Heptanal	0.704	2.166	1.779E-13	9.799E-12
193.1223	$C_{12}H_{16}O_2$	2,6-Diisopropyl-1,4-quinone	0.693	1.714	3.978E-13	1.696E-11
157.1223	$C_9H_{16}O_2$	4-Hydroxynonenal	0.685	2.072	7.430E-13	2.889E-11
181.0082	$C_4H_2OF_6$	Sevoflurane	0.679	0.489	1.092E-12	4.009E-11
75.0804	$C_4H_{10}O$	1-Butanol	0.677	0.950	1.307E-12	4.641E-11
99.0804	$C_6H_{10}O$	3-Hexenal	0.654	0.955	6.240E-12	1.650E-10
95.0491	C_6H_6O	Phenol	0.646	1.430	1.048E-11	2.514E-10
183.1743	$C_{12}H_{22}O$	2-Dodecenal	0.629	3.668	3.006E-11	6.623E-10
224.0666	$C_9H_9O_4N_3$	/	0.610	1.372	9.666E-11	1.727E-09
97.1012	C_7H_{12}	/	0.556	3.533	1.852E-09	2.661E-08
77.0386	C_6H_4	/	0.545	1.322	3.198E-09	4.271E-08
151.0754	$C_9H_{10}O_2$	4-Hydroxy-3-methylacetophenone	0.540	2.743	3.990E-09	5.275E-08
165.1638	$C_{12}H_{20}$	/	0.537	3.354	4.653E-09	6.031E-08
81.0699	C_6H_8	/	0.533	1.265	5.770E-09	7.196E-08
139.1117	$C_9H_{14}O$	Nonadienal	0.524	1.047	8.734E-09	1.046E-07
298.0822	$C_{15}H_{11}O_4N_3$	/	0.524	1.937	8.785E-09	1.046E-07
185.1900	$C_{12}H_{24}O$	9-Dodecen-1-ol	0.520	3.036	1.101E-08	1.288E-07
71.0604	$C_3H_6N_2$	/	0.512	1.426	1.552E-08	1.739E-07
171.1743	$C_{11}H_{22}O$	2-Undecanone	0.508	3.194	1.893E-08	2.085E-07
297.0816	$C_{10}H_{16}O_{10}$	/	0.504	1.932	2.255E-08	2.416E-07
148.0968	$C_6H_{13}O_3N$	/	0.502	-2.270	2.569E-08	2.716E-07
299.0788	$C_{15}H_6N_8$	/	0.501	1.674	2.593E-08	2.721E-07
157.1587	$C_{10}H_{20}O$	7E-Decen-1-ol	0.487	2.653	5.003E-08	5.010E-07
79.0542	C_6H_6	Benzene	0.482	1.087	6.230E-08	6.146E-07
135.0804	$C_9H_{10}O$	Phenylacetone	0.471	2.631	1.015E-07	9.650E-07
75.0553	$C_2H_6ON_2$	/	0.469	1.263	1.085E-07	1.024E-06
85.0284	$C_4H_4O_2$	4-Hydroxybut-2-ynal	0.465	2.663	1.327E-07	1.218E-06
143.1430	$C_9H_{18}O$	Pelargonaldehyde	0.448	2.235	2.647E-07	2.258E-06
108.0808	C_7H_9N	Benzylamine	0.443	2.261	3.312E-07	2.736E-06
91.0542	C_7H_6	/	0.442	2.504	3.450E-07	2.798E-06
169.1587	$C_{11}H_{20}O$	/	0.428	3.998	6.103E-07	4.663E-06
70.0651	C_4H_7N	/	0.419	-0.818	8.731E-07	6.521E-06

141.0910	$C_8H_{12}O_2$	4-oxo-2-octenal	0.418	2.584	9.250E-07	6.870E-06
83.0855	C_6H_{10}	/	0.395	2.858	2.283E-06	1.564E-05
73.0284	$C_3H_4O_2$	/	0.390	3.679	2.710E-06	1.828E-05
133.1012	$C_{10}H_{12}$	/	0.386	3.634	3.177E-06	2.121E-05
173.1536	$C_{10}H_{20}O_2$	/	0.374	3.255	4.890E-06	3.169E-05
113.0961	$C_7H_{12}O$	/	0.363	1.613	7.535E-06	4.699E-05
117.0910	$C_6H_{12}O_2$	4-Hydroxyhexan-3-one	0.355	1.885	9.998E-06	6.119E-05
197.1536	$C_{12}H_{20}O_2$	Geranyl acetate	0.337	1.056	1.895E-05	1.113E-04
132.1019	$C_6H_{13}O_2N$	/	0.333	-0.724	2.189E-05	1.264E-04
105.0447	$C_6H_4N_2$	2-Cyanopyridine	0.332	1.450	2.225E-05	1.279E-04
180.0867	$C_6H_{13}O_5N$	/	0.331	-0.572	2.298E-05	1.315E-04
179.1389	$C_7H_{18}O_3N_2$	/	0.325	0.558	2.876E-05	1.604E-04
89.0233	$C_3H_4O_3$	Malonic semialdehyde	0.318	1.367	3.619E-05	2.002E-04
180.1594	$C_8H_{21}O_3N$	/	0.309	-0.863	5.020E-05	2.698E-04
186.1489	$C_{10}H_{19}O_2N$	/	0.301	-0.740	6.488E-05	3.364E-04
