# Supplementary material for: Breath Analysis of Propofol and Associated Metabolic Signatures: A Pilot Study Using Secondary Electrospray Ionization–High-resolution Mass Spectrometry
Source: Anesthesiology. 2025 Apr 21;143(2):345–56. doi: 10.1097/ALN.0000000000005531 (PMC12227210; doi:10.1097/ALN.0000000000005531)
Supplement: Supplementary file 8 [file aln-143-345-s008.pdf]

Table S3. Information of identified up-regulated compounds found in the breath data.

m/z	Adduct Form	Name	Chemical group	HMDB ID	PubChem ID	KEGG ID	Formular	p	q	Log2FC
75.0804	$[M+H]^+$	1-Butanol	Fatty alcohols	HMDB0004327	263	C06142	$C_4H_{10}O$	3.3E-06	1.3E-04	5.57
79.0542	$[M+H]^+$	Benzene	Benzene and substituted derivatives	HMDB0001505	241	C01407	C_6H_6	5.0E-03	1.1E-02	2.60
85.0284	$[M+H]^+$	4-Hydroxy-2-butenoic acid gamma-lactone	Furanones	HMDB0032330	10341	C17601	$C_4H_4O_2$	1.1E-04	1.4E-03	1.03
		4-Hydroxybut-2-ynal	Organooxygen compounds	/	439778	C02648				
89.0233	$[M+H]^+$	Malonic semialdehyde	Carbonyl compounds	HMDB0011111	868	C00222	$C_3H_4O_3$	1.5E-03	5.7E-03	1.49
93.0547	$[M+H]^+$	Glycerol	Monosaccharides	HMDB0000131	753	C00116	$C_3H_8O_3$	1.8E-02	2.7E-02	1.23
95.0491	$[M+H]^+$	Phenol	Benzene and substituted derivatives	HMDB0000228	996	C00146	C_6H_6O	2.2E-08	7.4E-06	2.49
99.0804	$[M+H]^+$	3-Hexenal	Fatty aldehydes	HMDB0031497	643139	C16310	$C_6H_{10}O$	1.8E-04	1.8E-03	3.26
		Cyclohexanone	Ketones	HMDB0003315	7967	C00414				
105.0447	$[M+H]^+$	2-Cyanopyridine	Pyridines	/	7522	C02221	$C_6H_4N_2$	3.9E-03	9.5E-03	1.74
108.0808	$[M+H]^+$	Benzylamine	Benzene and substituted derivatives	HMDB0033871	7504	C15562	C_7H_9N	2.2E-04	2.0E-03	1.18
		o-Toluidine	Benzene and substituted derivatives	HMDB0041965	7242	C14403				
		N-Methylaniline	Benzene and substituted derivatives	HMDB0255185	7515	C02299				
115.1117	$[M+H]^+$	Heptanal	Fatty aldehydes	HMDB0031475	8130	C14390	$C_7H_{14}O$	7.7E-06	2.2E-04	1.89
		2-Heptanone	Hydrocarbons	HMDB0003671	8051	C08380				

117.0910	$[M+H]^+$	4-Hydroxyhexan-3-one	Hydrocarbons	/	95609	C02948	$C_6H_{12}O_2$	3.4E-04	2.5E-03	1.08
119.0703	$[M+H]^+$	Ethyl lactate	Carboxylic acid esters	HMDB0040735	7344		$C_5H_{10}O_3$	2.2E-02	3.2E-02	1.13
121.0648	$[M+H]^+$	Acetophenone	Acetophenones	HMDB0033910	7410	C07113	C_8H_8O	1.3E-03	5.5E-03	1.13
		4-Hydroxystyrene	Benzene and substituted derivatives	HMDB0004072	62453	C05627				
		Phenylacetaldehyde	Phenylacetaldehydes	HMDB0006236	998	C00601				
135.0804	$[M+H]^+$	Phenylacetone	Benzene and substituted derivatives	/	7678	C15512	$C_9H_{10}O$	9.2E-04	4.5E-03	1.05
		Indan-1-ol	Indenes	HMDB0059601	22819	C01710				
137.1073	$[M+H]^+$	2,3-Diethylpyrazine	Diazines	HMDB0041253	27458	/	$C_8H_{12}N_2$	4.1E-02	4.8E-02	1.40
139.0754	$[M+H]^+$	3-Methoxybenzyl alcohol	Benzene and substituted derivatives	HMDB0034241	7738	/	$C_8H_{10}O_2$	6.0E-04	3.3E-03	1.41
		Tyrosol	Benzene and substituted derivatives	HMDB0004284	10393	C06044				
139.1117	$[M+H]^+$	Nonadienal	Fatty aldehydes	/	5283340	C16323	$C_9H_{14}O$	7.3E-04	3.9E-03	2.65
		2-Pentylfuran	Heteroaromatic compounds	HMDB0013824	19602	/				
141.0910	$[M+H]^+$	4-oxo-2-octenal	Fatty aldehydes	/	5362996	/	$C_8H_{12}O_2$	3.3E-04	2.5E-03	1.06
143.1430	$[M+H]^+$	Pelargonaldehyde	Fatty aldehydes	HMDB0059835	31289	/	$C_9H_{18}O$	1.3E-03	5.5E-03	1.29
151.0754	$[M+H]^+$	4-Hydroxy-3-methylacetophenone	Carbonyl compounds	/	70135	/	$C_9H_{10}O_2$	9.4E-06	2.5E-04	1.31
		4-Hydroxycoumarin	Flavonoids	HMDB0003654	5280535	C02646				
		2-Methoxy-4-vinylphenol	Benzene and substituted derivatives	HMDB0013744	332	C17883				
		2-Phenylpropionate	Phenylpropanoids	HMDB0011743	10296	/				

151.0754	$[M+H]^+$	Hydrocinnamic acid	Phenylpropanoids	HMDB0000764	107	C05629	$C_9H_{10}O_2$	9.4E-06	2.5E-04	1.31
155.1430	$[M+H]^+$	2-Decenal	Fatty aldehydes	HMDB0030999	5283345	/	$C_{10}H_{18}O$	3.3E-03	8.6E-03	1.26
157.0859	$[M+H]^+$	4,4-Diethoxybut-2-ynal	Organooxygen compounds	/	2724485	/	$C_8H_{12}O_3$	4.2E-02	4.8E-02	1.86
157.1223	$[M+H]^+$	4-Hydroxynonenal	Fatty aldehydes	HMDB0004362	5283344	/	$C_9H_{16}O_2$	1.1E-05	2.8E-04	1.87
157.1587	$[M+H]^+$	7E-Decen-1-ol	Fatty alcohols	/	5283293	/	$C_{10}H_{20}O$	9.4E-04	4.5E-03	1.22
		Decanal	Fatty aldehydes	HMDB0011623	8175	C12307				
		p-Menthan-4-ol	Isoprenoids	HMDB0035726	574674					
		Menthol	Isoprenoids	HMDB0003352	16666	C00400				
167.1795	$[M+H]^+$	trans-2-trans-4-Nonadiene	Hydrocarbons	HMDB0032537	5358327	/	$C_{12}H_{22}$	2.5E-02	3.4E-02	2.20
171.1743	$[M+H]^+$	2-Undecanone	Ketones	HMDB0033713	8163	C01875	$C_{11}H_{22}O$	3.4E-04	2.5E-03	1.08
179.1430	$[M+H]^+$	Propofol	Benzene and substituted derivatives	HMDB0014956	4943	C07523	$C_{12}H_{18}O$	7.1E-08	9.6E-06	4.26
181.0082	$[M+H]^+$	Sevoflurane	Sevoflurane fragmentation	HMDB0015366	5206	/	$C_4H_2OF_6$	1.5E-04	1.7E-03	7.81
183.1743	$[M+H]^+$	2-Dodecenal	Fatty aldehydes	HMDB0031020	5283361	/	$C_{12}H_{22}O$	4.8E-06	1.6E-04	1.11
185.1900	$[M+H]^+$	9-Dodecen-1-ol	Fatty alcohols	/	5283277	/	$C_{12}H_{24}O$	7.2E-04	3.9E-03	1.09
		Lauric aldehyde	Fatty aldehydes	HMDB0033933	8194	C02278				
191.1066	$[M+H]^+$	Prenyl benzoate	Prenol lipids	HMDB0032488	21265	C03885	$C_{12}H_{14}O_2$	4.4E-05	7.7E-04	2.36
193.1223	$[M+H]^+$	2,6-Diisopropyl-1,4-quinone	propofol metabolites	/	642540	/	$C_{12}H_{16}O_2$	3.7E-08	7.4E-06	2.58
197.1536	$[M+H]^+$	Geranyl acetate	Fatty esters	/	1549026	/	$C_{12}H_{20}O_2$	2.4E-02	3.4E-02	1.84
221.1900	$[M+H]^+$	2,6-Di-tert-butyl-4-methylphenol	Benzene and substituted derivatives	HMDB0033826	31404	C14693	$C_{15}H_{24}O$	1.5E-05	3.3E-04	1.15

221.1900	$[M+H]^+$	propofol isopropyl ether	Benzene and substituted derivatives	/	/	/	$C_{15}H_{24}O$	1.5E-05	3.3E-04	1.15
243.2682	$[M+H]^+$	14-Methyl-1-pentadecanol	Fatty alcohols	/	5283265	/	$C_{16}H_{34}O$	4.2E-03	9.8E-03	1.20
		1-Hexadecanol	Fatty alcohols	HMDB0003424	2682	C00823				

m/z: mass to charge ratio; HMDB: human metabolome database; KEGG: Kyoto Encyclopedia of Genes and Genomes database; Log2FC: Log2 fold change per features by taking the Log2 of the ratio of the breath measurement post-induction over pre-induction.
